# Body mass index links night work intensity with higher low-grade systemic inflammation: results from a field study in humans

**DOI:** 10.1007/s00420-026-02218-2

**Published:** 2026-07-14

**Authors:** Kirsten Nabe-Nielsen, Kyriaki Papantoniou, Anne Emily Saunte Fiehn Arup, Mette Sallerup, Helena Breth Nielsen, Vivi Schlünssen, Anne Helene Garde

**Affiliations:** 1 The National Research Centre for the Working Environment, Lersø Parkallé 105, 2100 Copenhagen, Denmark; 2 Department of Public Health, University of Copenhagen, Copenhagen, Denmark; 3 Department of Epidemiology, Center for Public Health, Medical University of Vienna, Vienna, Austria; 4 Barcelona Institute for Global Health (ISGlobal), Barcelona, Spain; 5 Department of Public Health, Research Unit for Environment, Danish Ramazzini Centre, Aarhus, Denmark

**Keywords:** Circadian disruption, Healthcare, Nurses, Systemic inflammation, Occupational exposure, Work schedule patterns, Biomarkers

## Abstract

**Objective:**

To examine effects of night work on low-grade systemic inflammation, measured as high-sensitivity C-reactive protein (hsCRP concentration), in hospital-employed women.

**Methods:**

We analyzed baseline data from 929 women in the 1001 nights-cohort. Seven self-reported metrics captured night work history (never, past, current), night work duration (years) and recency (time since last night shift), current schedule (e.g., permanent night or 2- or 3-shift work), and intensity (number of weekly and consecutive night shifts, and night shifts in the past six days). We measured hsCRP concentration in blood. Associations between night work and log-transformed hsCRP concentration were estimated using generalized linear models: Model 1 adjusted for age and education; Model 2 additionally for body mass index (BMI), blood pressure, and health behaviors; Model 3 further for sleep duration and quality.

**Results:**

Permanent night workers had a 49% higher hsCRP concentration than permanent day workers (estimate = 1.49; 95% CI: 1.07–2.10). In night shift workers, working ≥ 3 night shifts per week or ≥ 3 consecutive night shifts were associated with 36–53% higher hsCRP concentration compared with one weekly night shift or only single-night shifts. These associations were attenuated and became non-significant after adjustment for cardiometabolic risk factors. Additional analyses revealed that BMI largely attenuated the associations between night work intensity and hsCRP concentration. No associations were observed for other night work metrics.

**Conclusion:**

Permanent night work and higher night work intensity were associated with higher hsCRP concentration, with differences largely explained by BMI. Reducing night work intensity and targeting modifiable factors related to BMI may help reduce low-grade systemic inflammation among night workers.

**Supplementary material:**

The online version of this article (10.1007/s00420-026-02218-2) contains supplementary material, which is available to authorized users.

## Introduction

Circadian disruption, i.e. transient or chronic disturbances of the circadian system, occurs when 24-hour oscillations, found in essentially every physiological process in the human body, are disturbed (Vetter 2020). While several factors can disrupt circadian rhythms, including travel across time zones, social jet lag, and mistimed exposure to meals, sleep, and artificial light, night work remains the most significant and pervasive cause by inducing sustained misalignment between the internal biological clock and the external environment (Potter et al. 2016).

In Europe, 24% of the population work at night (Eurofound 2023). Night work causes circadian misalignment due to mistiming of the sleep/wake cycle with the solar day, and consequent exposure to light at night, and night work has been evaluated as probably carcinogenic to humans by the International Agency for Research in Cancer (IARC) (International Agency for Research on Cancer 2019). Increased inflammation could be a key mechanism linking night work with cancer: The day-oriented circadian organization actively suppresses inflammation through molecular mechanisms, neuroendocrine and autonomic gating of immune traffic, and gut rhythmicity (Castanon-Cervantes et al. 2010; Gibbs et al. 2013, Scheiermann et al. 2013). Yet, circadian misalignment removes these brakes and leads to chronically higher inflammation. The ability of inducing chronic inflammation is one of the key characteristics of carcinogenic agents in humans, due to inflammatory processes’ disruption of local tissue homeostasis, alterations of cell signaling, and activation of proto-oncogenes (Smith et al. 2016).

C-reactive protein (CRP) is an acute-phase inflammatory marker that is typically present at concentrations below 10 mg/L under normal conditions but may rise to levels exceeding 100 mg/L in response to bacterial infection or severe tissue injury. Low-grade systemic inflammation is characterized by CRP levels in the upper range of normal, reflecting a chronic, low-level inflammatory state rather than an acute response, and it does not have a universally defined threshold. Experimental studies in humans found that circadian misalignment increases low-grade systemic inflammation measured as high-sensitive CRP (hsCRP) concentration (Leproult et al. 2014; Morris et al. 2016, 2017; Wright et al. 2015). A few observational field studies have investigated how these short-term experimental studies translate into real-life shift workers compared with day workers (Puttonen et al. 2011; Skogstad et al. 2019, 2025; Velazquez-Kronen et al. 2023) or non-night workers (Johnson et al. 2020). These studies reported a higher hsCRP concentration in rotating shift workers (Velazquez-Kronen et al. 2023), an increase in hsCRP concentration among employees with a high night shift load (Skogstad et al. 2025) or more years with night shifts (Johnson et al. 2020; Skogstad et al. 2019), and higher hsCRP concentration in men (but not women) with 3-shift work schedules (Puttonen et al. 2011). Yet, evidence about acute and chronic effects of night work on low-grade systemic inflammation, including knowledge on reversibility and dose-response effects of relevant night work exposure metrics, such as duration and intensity (number of weekly or consecutive night shifts), is still lacking (Erdem et al. 2025). The aim of the current study was, therefore, to investigate the effects of night work on low-grade systemic inflammation measured as the hsCRP concentration in humans. Our overall hypothesis was that exposure to night work increases hsCRP concentration in a dose-response manner, but also that the effect might be reversible when ceasing night work.

## Methods

### Study design and inclusion criteria

We use baseline data from the 1001 nights-cohort (Nabe-Nielsen et al. 2025). Data for this cohort study were collected from September 2022 to April 2024. Participants were women employed in the Danish hospital sector. The baseline data collection consisted of clinical and interview data from the examination day and a baseline questionnaire. The recruitment and data collection are described in detail elsewhere (Nabe-Nielsen et al. 2025). Participation was voluntary and participants could withdraw from the project at any time.

### Participants

A total of 1075 individuals signed the informed consent form and were enrolled in the study. Participants were nurses, nursing assistants, midwives, medical doctors, biomedical laboratory scientists, and administrative staff employed either at a somatic or psychiatric hospital. The participants worked in a broad range of departments, e.g. medical and surgical wards as well as emergency and outpatient departments. The participants represented various medical specialties, including orthopedic surgery, gynecology and obstetrics, radiology, and oncology. Among the 1075 participants, 1038 provided a blood sample, and among these, 957 had information on at least one night work exposure variable and full information on the investigated covariates. We excluded 28 participants with hsCRP concentration > 10 mg/L as this indicates a recent infection (Ansar et al. 2013), and thus 929 participants were included in the analyses. Excluded participants had a slightly lower mean age (40.6 vs. 41.5 years) and were more frequently night workers (82.8% vs. 71.8%) compared with included participants.

### Exposure to night work

**Fig. 1:**
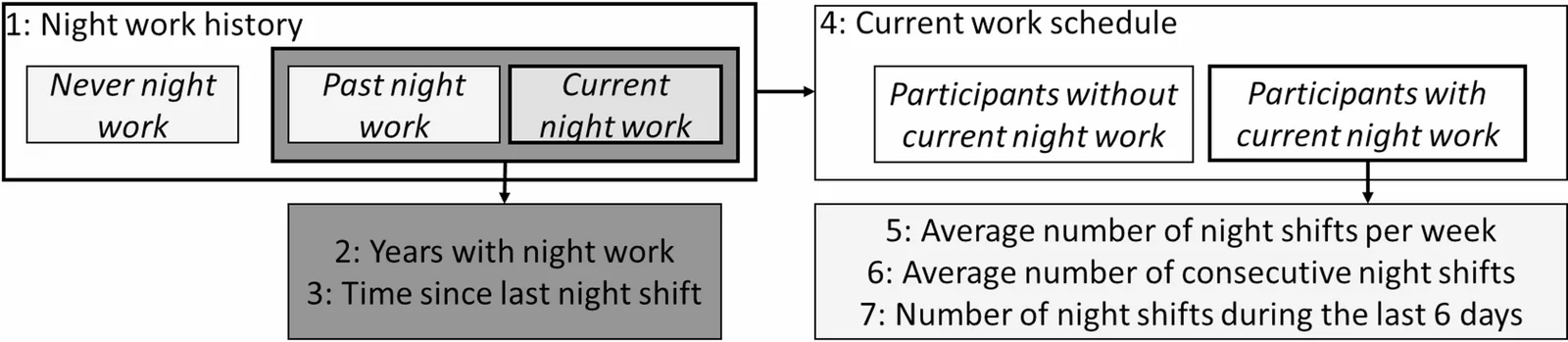
Night work exposure metrics used in the present study. The figure illustrates how the night work exposure metrics are interrelated, and the numbers refer to the variables described in the Methods section. All participants were eligible for analyses of night work history (**1**) and current work schedule (**4**). Only participants with past or current night work were used for the categorization of years with night work (**2**) and time since last night shift (**3**). Only participants with current night work (i.e. permanent night work or night work in combination with day and/or evening shifts) were used for the analyses of intensity measures, i.e. average number of weekly night shifts (**5**), average number of consecutive night shifts (**6**), and night shifts during the last 6 days (**7**)

We applied seven different night work exposure metrics (Fig. 1). All data on night shifts were self-reported and obtained either during the baseline examination or from the baseline questionnaire.

1. **Night work history**: Participants were categorized as “never” [ref], “past”, and “current” exposure to night work based on their response to (i) whether they had *ever* worked *at least three night shifts per month* (with night work defined as ≥ 3 h between 24:00 and 6:00 AM) and (ii) their current work schedule (see #4 below).
2. **Years with night work**: Among those reporting *ever* being exposed to night work (i.e. current or past night work), the total number of years with night work was used as a measure of cumulative exposure (categories: ≤1 [ref], > 1 to < 5 years, ≥ 5 to < 10 years, and ≥ 10 years).
3. **Time since last night shift**: Among past and current night workers the number of days/months/years since their last night shift was calculated using the reported date of their most recent night shift (categories: within past week, within past month, within past year, > 1 year ago [ref]). This was done in order to assess reversibility.
4. **Current work schedule**: Participants indicated their usual work schedule using predefined response options and were categorized as (a) permanent day workers [ref], (b) shift workers without night work (i.e., permanent evening or combined day–evening schedules), (c) shift workers with night work (i.e., day and/or evening shifts combined with night shifts), and (d) permanent night workers. To support accurate reporting, the questionnaire specified that day shifts typically occurred between 06:00 and 18:00, evening shifts between 15:00 and 24:00, and night shifts between 24:00 and 05:00. Participants were *not* provided with a specific timeframe for evaluating their usual schedule (e.g., the past six months).
5. **Average number of night shifts per week**: Among current night workers, we used the self-reported usual number of night shifts per week (categories: 1 [ref], 2, 3, ≥ 4).
6. **Average number of consecutive night shifts**: Among current night workers, we used the self-reported usual number of consecutive night shifts (categories: 1 [ref], 2, 3, ≥ 4).
7. **Number of night shifts during the last 6 days**: Among current night workers, we calculated the number of night shifts worked during the six days prior to the examination day as reported by the participants (categories: 0 [ref], 1, 2, 3, 4, ≥ 5).

### High-sensitive C-reactive protein

Up to 50 mL of blood was collected from each participant from the antecubital vein by venipuncture using Vacutainer tubes. Participants were non-fasting prior to sampling, and sampling was conducted between 7:30 and 17:00. 2 mL of blood from each participant was kept as whole blood. The rest of the blood underwent centrifugation for 15 min at 4000 revolutions per minute usually within 60–90 min after sampling. Afterwards, the samples were segregated into serum, EDTA plasma, and fraction (white blood cells and platelets), and stored in 1 mL aliquots at −80 °C.

The ABX Pentra CRP CP assay (licensed under USP 6,248,597, USP 6,828,158, and equivalent international patents) is a latex-enhanced immunoturbidimetric method designed for the precise quantification of CRP in serum and plasma samples. In this assay, CRP in the sample reacts with latex-sensitized anti-CRP antibodies, leading to agglutination. This agglutination induces a change in absorbance, which is directly proportional to the hsCRP concentration. The hsCRP concentration is then determined by interpolating the absorbance change against a calibration curve generated from reference calibrators of known concentrations. In total, 16 participants had a value below the limit of detection of 0.12 mg/L. To keep these participants in the analyses, the value below the minimal detection level was replaced with a random number drawn from a normal distribution with a mean of 0.067 mg/L and a standard deviation of 0.014 mg/L.

### Covariates

Decisions about inclusion of covariates were informed by previous reviews on hsCRP concentration and cardiovascular risk factors and education, respectively (Kaptoge et al. 2010; Muscatell et al. 2020) and comparable studies of night-work related circadian disruption and inflammatory markers (Johnson et al. 2020; Kwak et al. 2019; Puttonen et al. 2011; Velazquez-Kronen et al. 2023).

We included information about *age* on the date of enrolment. Height and weight for the calculation of *body mass index* (BMI) were obtained by trained staff at the examination day. Systolic and diastolic *blood pressure* were also measured at the examination day.

Information about education, health behaviors, and sleep was obtained from the baseline questionnaire and consisted of *duration of education* (6 response options ranging from compulsory schooling to long-cycle higher education program, more than 4 years), *smoking* (current, ex-smoker, never), *alcohol consumption* (units per week), and *leisure-time physical activity* (physically inactive; light physical activity 2–4 h per week; light physical activity > 4 h per week or vigorous physical activity 2–4 h per week; vigorous physical activity > 4 h per week or regularly high intensity exercise or competitions several times per week). The two latter response options were combined in the analyses. Participants also reported their *sleep quality* (excellent; very good; good; less good; bad) and *average sleep duration* in the last four weeks (5 h or fewer; 6 h; 7 h; 8 h; 9 h; 10 h).

We assessed chronotype by calculating a morningness-eveningness score (MEQ-score) based on the Morningness-Eveningness Questionnaire, with a higher score indicating more “morningness” (Horne et al. 1976). Information on chronic diseases (myocardial infarction, cardiac arrhythmia, stroke, high cholesterol, hypertension, chronic bronchitis, asthma, diabetes and cancer) was based on information from the background questionnaire “*Has your general practitioner ever told you suffered from any of these diseases”*, with the response options: “Yes”, “no”, and “don’t know”. For each participant, we calculated the sum of chronic diseases. As the majority had none of these chronic diseases the variable was dichotomized into 0 vs. ≥1 chronic disease. We used self-reported information about recent infections from the baseline questionnaire *“Having been bothered by having a cold within the past 4 weeks*”, with response options ranging from “not at all” to “very much”.

### Statistical analyses

First, we present the distribution (N, % and mean, SD) of covariates across current work schedule-groups and the median and the interquartile range (IQR) of the hsCRP concentration across covariates included in the present study. Second, as we modelled associations with a continuous outcome (hsCRP concentration), we used a generalized linear model to analyze the association of the seven exposure metrics (1–7) with log-transformed hsCRP concentration as outcome. HsCRP was log-transformed due to its non-normal distribution, characterized by a pronounced right skew. Estimates were back-transformed and results are presented as percentage change with their 95% confidence intervals (CI). RStudio version 4.5.1 was used for all analyses.

We adjusted for potential confounding factors in three steps: *Model 1* was adjusted for age and duration of education (main model), *Model 2* was further adjusted for cardiometabolic risk factors (smoking, alcohol consumption, physical activity, BMI, and blood pressure), while *Model 3* was further adjusted for sleep quality and sleep duration.

We conducted additional analyses (A–D) to further examine the relationships between the night work exposure metrics and hsCRP, and sensitivity analyses (E–F) to assess the robustness of our findings:

(A) For statistically significant night work exposure variables in Model 1, we reran the analyses while including the cardiometabolic risk factors one by one to assess which of them had the largest influence on the night work-hsCRP association (*post hoc analyses)*.
(B) Based on the findings from the additional analyses (A), we ran an analysis in which we estimated the proportion of the observed association between the selected night work exposure metrics and hsCRP that was explained by BMI. Mediation analyses were conducted using the counterfactual framework. The indirect (mediated) effect through BMI (Average Causal Mediation Effect, ACME), the average direct effect (Average Direct Effect, ADE), and the total effect were estimated using linear regression models for the mediator and outcome, adjusted for age and education. The proportion mediated was calculated as the ratio of the indirect effect to the total effect (ACME/total effect) and expressed as a percentage *(post hoc analyses)*.
(C) Adjusting for age and education, we examined the associations between selected night work metrics (the number of weekly and consecutive night shifts) in a sample restricted to shift workers with night work in combination with day/evening shifts, thereby excluding permanent night workers Additionally, we ran the mediation analyses and estimated ACME, ADE and the total effect in this sample (*post hoc analyses)*. We did this, as we observed that permanent night workers as a group exhibited a poorer health profile than other shift workers with night work. In addition, previous research suggests that the selection into permanent night work may be stronger than into other work schedule groups.
(D) We investigated if chronotype interacted with the night work exposure metrics on hsCRP concentrations with adjustment for age and education.
(E) We additionally adjusted for chronic diseases in Model 1 to investigate the influence of concurrent morbidity.
(F) We reran Model 1 in a sample restricted to those without a recent infection (i.e. reporting having had a cold) to separate recent acute infections from chronic low-grade systemic inflammation.

## Results

### Descriptive analyses

In this field study of 929 women, permanent night workers were older, had a shorter education, a higher prevalence of adverse health behaviors, more chronic diseases as well as shorter sleep duration, and lower sleep quality than the groups of shift workers (with or without night work) and permanent day workers. The group of shift workers with night work were younger, but did not otherwise differ substantially from shift workers without night work or permanent day workers (Table 1).

**Table 1:** Description of the study population across current work schedule-groups

	Permanent day workers				Shift workers without night^1^				Permanent night workers				Shift workers with night^2^				Total population			
	*N*	%	Mean	SD	*N*	%	Mean	SD	*N*	%	Mean	SD	*N*	%	Mean	SD	*N*	%	Mean	SD
Number (n)	175	18.8			87	9.4			60	6.5			607	65.3			929	100.0		
Age	175		44.6	10.8	87		45.4	13.3	60		46.9	10.8	607		39.5	11.7	929		41.5	12.0
Education (duration: ≥3 years)	148	84.6			65	74.7			42	70.0			532	87.6			787	84.7		
Smoking																				
Current	18	10.3			11	12.6			11	18.3			80	13.2			120	12.9		
Ex	50	28.6			34	39.1			23	38.3			198	32.6			305	32.8		
Never	107	61.1			42	48.3			26	43.3			329	54.2			504	54.4		
Alcohol (units per week)																				
0	49	28.0			37	42.5			32	53.3			193	31.8			311	33.5		
> 0–4	97	55.4			40	46.0			21	35.0			334	55.0			492	53.0		
> 4	29	16.6			10	11.5			7	11.7			80	13.2			126	13.6		
Physical activity																				
Sedentary	16	9.1			8	9.2			7	11.7			60	9.8			91	9.8		
Light	70	40.0			31	35.6			22	36.7			215	35.4			338	36.4		
Moderate/vigorous	89	50.9			48	55.2			31	51.7			332	54.7			500	53.8		
BMI	175		26.0	4.9	87		26.5	4.9	60		29.5	6.2	607		26.4	5.0	929		26.5	5.1
Blood pressure																				
Diastolic	175		79.2	9.7	87		78.7	9.3	60		82.9	9.9	607		77.7	9.3	929		78.4	9.5
Systolic	175		117.0	15.5	87		116.0	14.2	60		121.	13.1	607		114.0	13.1	929		115.0	13.8
Self-reported sleep quality																				
Excellent/very good	45	25.7			13	14.9			8	13.3			113	18.6			179	19.3		
Good	84	48.0			35	40.2			26	43.3			286	47.1			431	46.4		
Less good/bad	46	26.3			39	44.8			26	43.3			208	34.3			319	34.3		
Average hours of sleep last 4 weeks																				
≤ 6 h	56	32.0			46	52.9			37	61.7			270	44.5			409	44.0		
7 h	77	44.0			28	32.2			16	26.7			257	42.3			378	40.7		
≥ 8 h	42	24.0			13	14.9			7	11.7			80	13.2			142	15.3		
MEQ-score	175		57.2	7.7	87		53.6	6.9	59		45.8	8.9	606		52.5	7.7	927		53.1	8.2
Chronic diseases																				
None	117	66.9			54	62.1			24	40.0			418	69.3			613	66.3		
≥ 1	58	33.1			33	37.9			36	60.0			185	30.7			312	33.7		

^1^ Permanent evening or day/evening shifts

^2^ Day/night shifts, evening/night shifts or day/evening/night shifts.

The median hsCRP concentration was highest in permanent night workers (median = 1.5 mg/L; IQR = 3.0) and among those with ≥ 4 shifts per week (median = 1.5; IQR = 2.8). For other exposure categories the median level varied between 0.6 and 1.3 (Table 2). The highest hsCRP concentration was observed in participants who were older or sedentary and had a short duration of education, a high BMI, or high blood pressure (supplementary table A1).

**Table 2:** Description of the median hsCRP concentration and Inter Quartile Range (IQR) across exposure categories in the study population (*n* = 929)

	*N*	Median mg/L	IQR mg/L
Night work history ^a^			
Never	134	0.8	1.5
Past	113	0.7	1.7
Current	667	0.9	1.9
Missing	15		
Years with night work ^b^			
*≤* 1	79	0.9	2.0
> 1–<5	175	0.8	1.7
≥ 5–<10	149	1.0	2.1
≥ 10	350	0.9	1.8
Missing	27		
Time (years) since last night shift ^b^			
Within past week	379	0.9	2.0
Within past month	236	0.9	1.6
Within past year	67	1.1	2.4
> 1 year ago	64	0.9	1.7
Missing	34		
Current work schedule ^c^			
Permanent day	175	0.7	1.8
Shift workers w/o night	87	0.9	1.5
Permanent night	60	1.5	3.0
Shift workers w night	607	0.9	1.8
Missing	0		
Average number of night shifts per week ^d^			
1	272	0.9	1.5
2	228	0.9	2.0
3	74	1.1	2.9
4 or more	66	1.5	2.8
Missing	27		
Average number of consecutive night shifts ^d^			
1	91	0.6	1.4
2	183	0.9	1.7
3	243	1.1	2.3
4 or more	123	1.1	2.5
Missing	27		
Number of night shifts during the last 6 days ^d^			
0	332	0.9	1.8
1	123	1.0	1.8
2	85	0.9	2.8
3	77	0.8	1.2
4	36	1.3	3.3
5 or more	14	0.8	1.4
Missing	0		

^(a)^ Among all participants with available data; ^(b)^ Among ever night workers (past and current); ^(c)^ Among current night workers

### Main analyses

When adjusting for age and education (Model 1), we found that the hsCRP concentration in permanent night workers was 49% higher than in permanent day workers (estimate = 1.49; 95% CI: 1.07–2.10). In the whole group of night workers, those with 3 or ≥ 4 weekly night shifts or 3 or ≥ 4 consecutive night shifts had 36–53% higher hsCRP concentration compared with those with one night shift per week or working only single-night shifts (Table 3). Adjustment for cardiometabolic risk factors attenuated all associations, yet, in Model 2 and 3, working 3 night shifts in the week leading up to the examination day was associated with a significantly lower hsCRP concentration (Model 2: estimate = 0.77; 95% CI: 0.59–0.99; Model 3: estimate = 0.76; 95% CI: 0.59–0.99).

**Table 3:** The association between night work exposure metrics and hsCRP concentration. Results are presented as relative differences in the mean concentrations with their lower (L) and upper (U) 95% confidence intervals (CI)

	Model 1			Model 2			Model 3		
	Estimate	L95%CI	U95%CI	Estimate	L95%CI	U95%CI	Estimate	L95%CI	U95%CI
Night work history									
Never	1.00			1.00			1.00		
Past	1.04	0.78	1.39	1.09	0.85	1.41	1.10	0.85	1.42
Current	1.19	0.96	1.48	1.13	0.93	1.36	1.14	0.94	1.38
Years with night work									
*≤* 1	1.00			1.00			1.00		
> 1–<5	0.94	0.69	1.28	1.01	0.77	1.32	1.01	0.77	1.33
*≥* 5-<10	0.99	0.72	1.37	1.07	0.81	1.43	1.08	0.81	1.44
*≥* 10	0.96	0.69	1.34	1.01	0.75	1.35	1.02	0.76	1.37
Time since last night shift									
Within past week	1.00			1.00			1.00		
Within past month	0.97	0.80	1.17	1.04	0.88	1.23	1.05	0.89	1.24
Within past year	0.97	0.71	1.31	1.07	0.82	1.39	1.06	0.82	1.39
> 1 year ago	0.95	0.70	1.30	0.99	0.76	1.30	1.00	0.76	1.31
Current work schedule									
Permanent day	1.00			1.00			1.00		
Shift work w/o night	1.04	0.78	1.40	1.02	0.79	1.33	1.03	0.79	1.34
Permanent night	**1.49**	**1.07**	**2.10**	1.05	0.78	1.42	1.06	0.78	1.43
Shift work w night	1.14	0.94	1.49	1.08	0.91	1.28	1.09	0.92	1.30
Average number of night shifts per week									
1	1.00			1.00			1.00		
2	1.19	0.97	1.46	1.02	0.85	1.22	1.01	0.84	1.22
3	**1.36**	**1.01**	**1.83**	1.03	0.79	1.36	1.03	0.78	1.35
4 or more	**1.50**	**1.10**	**2.06**	1.14	0.85	1.52	1.12	0.83	1.51
Average number of consecutive night shifts									
1	1.00			1.00			1.00		
2	1.20	0.89	1.60	1.06	0.81	1.38	1.04	0.80	1.36
3	**1.53**	**1.15**	**2.02**	1.29	0.99	1.66	1.28	0.99	1.66
4 or more	**1.46**	**1.06**	**2.00**	1.12	0.84	1.50	1.11	0.83	1.48
Average number of night shift during the last 6 days									
0	1.00			1.00			1.00		
1	1.03	0.81	1.31	0.98	0.79	1.22	0.98	0.79	1.21
2	0.93	0.71	1.23	0.87	0.68	1.11	0.85	0.66	1.09
3	0.80	0.60	1.06	**0.77**	**0.59**	**0.99**	**0.76**	**0.59**	**0.99**
4	1.23	0.83	1.84	1.18	0.82	1.68	1.14	0.80	1.64
5 or more	1.02	0.55	1.89	0.95	0.55	1.65	0.94	0.54	1.64

Model 1: Adjusted for age and education

Model 2: Adjusted for age, education, smoking, alcohol consumption, physical activity, BMI, and blood pressure. Model 3: Adjusted for age, education, smoking, alcohol consumption, physical activity, BMI, and blood pressure, sleep duration and sleep quality

Significant differences are shown in bold

### Additional and sensitivity analyses (A-F)

To further explore the influence of the cardiometabolic risk factors adjusted for in Model 2, we added them one by one to the statistical model (A, Supplementary Table A2). Adjusting for BMI systematically attenuated all estimated associations between night work exposure metrics and hsCRP concentration, whereas the remaining factors only had a negligible influence on the observed associations. When further exploring mediation by BMI, we found that 66% (95% CI: 30–214%) of the association between ≥ 4 weekly night shifts and hsCRP was mediated by differences in BMI. The corresponding results for 3 and ≥ 4 consecutive night shifts were 52% (95% CI: 25–135%) and 74% (95% CI: 35–237%), respectively (B, Supplementary Table A3).

In a sample restricted to shift workers with night work (C, Supplementary tTable A4), we found that more weekly night shifts and more consecutive night shifts were significantly associated with higher hsCRP concentration (≥ 4 weekly night shifts: estimate = 1.69, 95% CI = 1.03–2.76; 3 consecutive night shifts: estimate = 1.48, 95% CI = 1.11–1.96; ≥4 consecutive night shifts: estimate = 1.41; 95% CI: 1.001–1.99). The mediation analyses in this subsample confirmed the mediated proportion found in the total sample mainly for “consecutive night shifts”, whereas these estimates were all statistically insignificant for “night shifts per week” (Supplementary Table A5).

We found no evidence of heterogeneity of the association of night work with hsCRP concentration, i.e. no interaction with chronotype (D). Finally, adjusting the results of Model 1 for chronic diseases (E) or restricting the sample to those without an infection during the past 4 weeks (F), did not influence the estimates (D, E and F are not presented in tables).

## Discussion

### Main findings

This large study of Danish female hospital workers, found that permanent night workers, compared with permanent day workers, had a higher hsCRP concentration. Furthermore, among night workers, working three or more weekly or consecutive night shifts was associated with higher hsCRP concentration compared with single-night shifts. However, in *post hoc* analyses the associations between night work and hsCRP concentration largely disappeared after adjustment for cardiometabolic risk factors, with further mediation analyses indicating that higher BMI accounted for about 50–70% of the observed associations. Importantly, however, given the cross-sectional design, BMI may act as both a confounder and a mediator, and these roles are not mutually exclusive.

### Comparison with previous findings

Compared with day work, night work involves circadian misalignment, sleep disruption, and altered timing of key behaviors. This context provides a framework for interpreting the associations observed in the present study. Our study is among the first to distinguish permanent night workers from rotating shift workers with night work, and to examine exposure metrics reflecting night work intensity, defined by the average number of weekly and consecutive night shifts. The results suggest that permanent a night work and a higher night work intensity is linked to a higher hsCRP concentration, particularly three or more weekly/consecutive night shifts. These findings support current recommendations of a maximum of three consecutive nights to prevent cancer (Garde et al. 2020). The large relative differences should be interpreted in light of the modest absolute differences in median hsCRP-levels, for example, 0.7 mg/L among permanent day workers versus 1.5 mg/L among permanent night workers, and in the context of prior evidence showing only a small relative increase in overall cancer incidence (HR = 1.02; 95% CI: 1.01–1.02) per 1 mg/L increase in hsCRP concentration (Zhu et al. 2022).

In an experimental study of three male and six female experienced shift workers, the hsCRP concentration increased by 11% after one simulated night shift compared with simulated day shifts (Morris et al. 2017). In another study of 14 healthy male and female adults, hsCRP concentration rose by 7% after one 24-hour period of circadian misalignment, but did not increase further with additional days of misalignment (up to three days) (Morris et al. 2016). A third experimental study including 26 healthy adults examined the separate effects of sleep restriction and circadian misalignment and found that three days of misaligned and shortened sleep significantly increased hsCRP concentration, whereas sleep restriction alone did not (Leproult et al. 2014). Finally, an experimental study among 17 men and women showed that hsCRP concentration increased during scheduled wakefulness under a circadian misalignment condition; however, plasma concentrations of both pro- and anti-inflammatory markers rose, indicating that participants did not shift to an overall pro-inflammatory state (Wright et al. 2015). Collectively, these experimental studies provide strong mechanistic evidence for the acute effects of circadian misalignment, as seen in night work, on hsCRP concentration, but their findings need to be integrated with evidence from real-world night workers.

In comparable observational cross-sectional field studies of predominantly female hospital employees, two studies found no association between self-reported rotating night shift work, compared with day work (Jordakieva et al. 2022) or self-reported number of night shifts during the preceding two weeks (Johnson et al. 2020) and hsCRP concentration. The latter finding is consistent with our results regarding the number of night shifts during the past six days and days since the last night shift. In descriptive analyses, one study reported higher hsCRP concentration in both former and current shift workers (Rizza et al. 2020), and also studies adjusting for health behaviors found associations between the hsCRP concentration and night shift work (Nikpour et al. 2019; Pavanello et al. 2017) as well as years of rotating night shifts (Johnson et al. 2020). Thus, our findings are only partly consistent with previous studies, as we observed a higher hsCRP concentration only among permanent night workers and those with more weekly or consecutive night shifts, but not in former night workers or with increasing years of night work. Notably, the group differences disappeared after adjustment for cardiometabolic risk factors, particularly BMI. While selection into night work is possible (Daghlas et al. 2021), obesity is also a plausible consequence of night work (Boini et al. 2022), which may arise from both the metabolic effects of circadian misalignment and sleep deprivation (Chaput et al. 2023). In addition, behavioral factors such as altered food preferences when fatigued and contextual factors, including limited access to healthy food during night shifts, may also contribute (McIntosh et al. 2023). These factors are likely bidirectionally related to night work, functioning both as confounders and mediators, such that adjusting for them may introduce overadjustment and attenuate associations. Notably, only BMI, rather than other socially stratified health behaviors such as smoking and leisure-time physical activity, accounted for the observed association between night work intensity and hsCRP concentration.

The mixed findings from hospital-based studies are echoed in research on shift workers from other sectors. Female employees following a 4 day shifts-2 days off-4 night shifts-2 days off rotation at an electronics manufacturing company had higher hsCRP concentration after extensive adjustment for confounders, including age, waist circumference, BMI, marital status, education, smoking, blood pressure, cholesterol, and triglycerides (Kwak et al. 2019). In contrast, female two-shift workers (no nights) from an airline company had a higher hsCRP concentration than day workers, but this difference was not statistically significant after full adjustment for age, infection, smoking, alcohol, physical activity, education, and obesity (Puttonen et al. 2011). In the same study, however, male three-shift workers had a significantly higher hsCRP concentration than day workers after adjustment (Puttonen et al. 2011). The two-shift workers were primarily engaged in maintenance, storage, and customer service, whereas the three-shift workers performed maintenance and cargo handling (Puttonen et al. 2011). In industrial samples of male shift workers, alternating weekly between day shifts (08:00–17:00) and night shifts (16:30–03:00/04:00) at an automotive company, shift work was associated with higher hsCRP concentration both before (Ye et al. 2020) and after adjustment for age, BMI, waist circumference, alcohol consumption, smoking, exercise, sleep, education, and weekly working hours (Kim et al. 2016). The finding of a higher hsCRP concentration among female employees in electronics manufacturing was later replicated in a partly overlapping population including both males and females (Woo et al. 2022). Other studies in industrial settings have reported heterogeneous results: One observed an insignificant decrease in hsCRP concentration after a plant shutdown among shift workers with 8- or 12-hour night shifts (Skogstad et al. 2022), while others found higher hsCRP concentration with increasing years of shift work (Skogstad et al. 2019), or over six years of follow-up after adjustment for age, sex, and smoking (Skogstad et al. 2025).

Population-based studies have also produced inconsistent findings. One study using crude measures of night work exposure found no difference in hsCRP concentration between day workers and shift workers with or without night work, or permanent night workers (Amano et al. 2018). In contrast, another study applying a more detailed exposure assessment reported higher hsCRP concentration in former and rotating shift workers, yet the cumulative dose of night work exposure could not be determined (Velazquez-Kronen et al. 2023). Furthermore, stratified analyses revealed that certain shift work characteristics were associated with higher hsCRP concentration primarily among white men, specifically, being a former shift worker, working afternoon shifts, and having shift work tenure of 1–10 or ≥ 10 years (Velazquez-Kronen et al. 2023).

Differences in covariate adjustment alone do not appear to explain the inconsistencies between studies, as some have reported associations between night or shift work and hsCRP concentration even after extensive adjustment for cardiometabolic risk factors, including BMI or obesity. Therefore, a unified model describing the interplay between shift work, markers of systemic inflammation, and cardiometabolic health across populations has yet to be established.

In a recent systematic review, 42 studies on shift work and inflammatory markers were identified. In a meta-analysis of 15 of the studies on CRP specifically, a standardized mean difference of 0.14 higher CRP concentration was observed among night shift workers compared with day workers (Erdem et al. 2025). Notably, a high risk of publication bias was identified, with generally small sample sizes (11 studies included fewer than 200 participants) and predominantly null findings in larger study populations. Thus, the present study contributes to the evidence base of larger studies, with analytical sample sizes of 600–900 participants. In line with the interpretation of our findings, the authors of the review highlight that the association between night shift work and elevated inflammation may be partly driven by indirect pathways, particularly through changes in lifestyle patterns among night workers (Erdem et al. 2025). This interpretation is consistent with our results, where adjustment for cardiometabolic risk factors, especially BMI, substantially attenuated the observed associations between night work intensity and hsCRP concentration.

### Strengths and limitations

The main strengths of this study include its large sample size, the detailed exposure assessment capturing both long-term and short-term night work exposure, and the comprehensive adjustment for multiple potential confounders. The current study aimed specifically to investigate the effects of night work-related circadian misalignment on hsCRP, and therefore we did not investigate other shift schedule characteristics, such as days off between shifts, early morning shifts, shift length, etc.

The cross-sectional design limits causal inferences of the effects and the ability to assess temporality, particularly between exposure and potential mediators such as health behaviors. Consequently, adjustment for factors such as smoking, BMI, and blood pressure may represent overadjustment when estimating associations between night work and hsCRP concentration, as these factors could be mediators in the exposure-outcome relation. Thus, our findings may reflect both causal effects and health-related selection into night work (Daghlas et al. 2021; Härmä et al. 2024), particularly if night workers with chronic diseases were more likely to participate in the cohort. Unfortunately, we lacked information on specific inflammatory diseases (or medical treatment of these) such as arthritis or inflammatory bowel disease, which could increase systemic low-grade systemic inflammation. Indeed, in our sample, permanent night workers reported more chronic diseases, which may reflect such selection as well as possible health effects of night work. However, sensitivity analyses with additional adjustment for the presence of specific chronic diseases did not materially affect the results.

Although we applied several exposure metrics, they were based on self-reported data, which may have introduced recall bias, particularly regarding the date of the last night shift, which for some participants could have been months or years earlier. Furthermore, the available information to define past night work (whether they had *ever* worked *at least three night shifts per month*) did not allow us to construct a detailed metric of previous exposure to night work. However, a Finnish study of hospital employees found that 96% of those classified as shift workers with night work according to payroll data also identified themselves as such in self-reported data (Härmä et al. 2017) supporting the use of self-reported exposure measures, when objective data are not available. In contrast, a recent study reported that the accuracy of lifetime night work exposure differed between breast cancer cases (86.2%) and controls (80.6%), suggesting possible bias (Vestergaard et al. 2024). As our study participants are not aware of the outcome, we expect any misclassification in our study to be non-differential with respect to the outcome, likely attenuating rather than generating spurious associations.

The finding of a lower hsCRP concentrations in the fully adjusted model among participants with three night shifts prior to enrolment, compared with those with no recent night shifts, was unexpected. One possible explanation is that this result reflects a chance finding arising from multiple comparisons. Furthermore, no consistent pattern was observed across adjacent exposure categories. In addition, the direction of the association is not biologically plausible and is inconsistent with experimental evidence on the acute effects of circadian misalignment on inflammatory markers, as well as with the overall pattern observed across our other analyses of night work intensity. Therefore, this result should be interpreted with caution.

### Generalizability

Similar associations have been reported among employees in other sectors, including industry and manufacturing, nursing homes, and airline companies (Kim et al. 2016; Kwak et al. 2019; Nikpour et al. 2019; Puttonen et al. 2011), as well as in the general working population (Velazquez-Kronen et al. 2023). However, the cardiometabolic risk profile of specific populations may influence both the degree of systemic low-grade systemic inflammation and the long-term health consequences of night work exposure. Although current evidence does not strongly support a sex-specific effect of night work on systemic low-grade systemic inflammation, findings from male-only studies and sex-stratified analyses suggest that the inflammatory response to night work may be more pronounced in men than in women (Kim et al. 2016; Puttonen et al. 2011; Velazquez-Kronen et al. 2023). Yet, in the previously mentioned literature review and meta-analysis, higher CRP-levels among female night workers were identified (Erdem et al. 2025). Thus, if there is indeed a sex difference, this may either have a biological underpinning or be due to sex-segregation in the labor market with males undertaking jobs encompassing a higher dose of night work (The Danish Working Environment Authority 2023). This interpretation remains speculative and cannot be confirmed by our data, but sex-specific effects could contribute to the discrepancy between our and earlier results in mixed samples, and extrapolation of our findings to male workers should be made with caution.

## Conclusion

This study found that permanent night work was associated with a higher hsCRP concentration, indicating increased systemic low-grade systemic inflammation. Higher night work intensity in terms of either ≥ 3 weekly night shifts or ≥ 3 consecutive night shifts were associated with an approximately 50% higher concentration of hsCRP concentration compared with working only one weekly night shift or only single-night shift. These associations were largely attenuated after adjustment for BMI. This suggests that cardiometabolic health, especially overweight and obesity are related to the inflammatory response in night workers, potentially acting as a mechanistic pathway (i.e., a mediator) or as a confounder, roles that are not mutually exclusive. While the cross-sectional design limits causal inference, the findings point to potential explanations of the link between intensive night work schedules and developing low-grade systemic inflammation. Thus, our findings suggest that preventive efforts may benefit from both optimizing work schedules to reduce night work intensity and implementing workplace interventions that promote conditions conducive to maintaining a healthy weight and preventing obesity in night shift workers.

## Supplementary material

Below is the link to the electronic supplementary material.

Supplementary file 1 (DOCX 19 kb)

## Data Availability

The data underlying this study are not publicly available due to data protection regulations under the General Data Protection Regulation (GDPR). Access to the data can be granted upon reasonable request and subject to approval by the data owners. Data analyses must be conducted by researchers physically located in Denmark and in collaboration with the study investigators. Interested researchers may contact the corresponding author for further information regarding data access procedures.
