# Supplementary material for: Body mass index links night work intensity with higher low-grade systemic inflammation: results from a field study in humans
Source: Int Arch Occup Environ Health. 2026 Jul 14;99(6):38. doi: 10.1007/s00420-026-02218-2 (PMC13369650; doi:10.1007/s00420-026-02218-2)
Supplement: Supplementary file 1 — (DOCX 19 kb) [file 420_2026_2218_MOESM1_ESM.docx]

| Supplementary Table A1: Description of the median hsCRP concentration and Inter Quartile Range (IQR) across categories of covariates in the study population (n=929). | | | | |
| --- | --- | --- | --- | --- |
|  | N |  | Median | IQR |
| Age |  |  |  |  |
| ≤29 | 217 |  | 0.9 | 2.0 |
| 30-39 | 220 |  | 0.8 | 1.6 |
| 40-49 | 223 |  | 0.7 | 1.4 |
| 50-59 | 194 |  | 1.2 | 2.0 |
| ≥60 | 75 |  | 1.3 | 2.5 |
|  |  |  |  |  |
| Education |  |  |  |  |
| <3 years | 142 |  | 1.4 | 2.7 |
| ≥3 years | 787 |  | 0.9 | 1.7 |
|  |  |  |  |  |
| Smoking |  |  |  |  |
| Current | 120 |  | 0.8 | 2.3 |
| Ex | 305 |  | 0.9 | 1.8 |
| Never | 504 |  | 0.9 | 1.7 |
|  |  |  |  |  |
| Alcohol (units per week) |  |  |  |  |
| 0 units | 311 |  | 1.1 | 2.4 |
| >0-4 | 492 |  | 0.8 | 1.5 |
| >4 | 126 |  | 0.7 | 1.6 |
|  |  |  |  |  |
| Physical activity |  |  |  |  |
| Sedentary | 91 |  | 1.4 | 2.7 |
| Light | 338 |  | 1.2 | 2.2 |
| Moderate/vigorous | 500 |  | 0.7 | 1.3 |
|  |  |  |  |  |
| BMI |  |  |  |  |
| ≤24.9 | 422 |  | 0.5 | 0.8 |
| 25.0-29.9 | 305 |  | 1.0 | 1.9 |
| 30.0-34.9 | 140 |  | 1.9 | 2.4 |
| ≥35.0 | 62 |  | 3.5 | 3.0 |
|  |  |  |  |  |
| Blood pressure |  |  |  |  |
| Diastolic <90 | 818 |  | 0.9 | 1.8 |
| Diastolic ≥90 | 111 |  | 1.2 | 2.1 |
| Systolic <140 | 875 |  | 0.9 | 1.9 |
| Systolic ≥140 | 54 |  | 1.4 | 1.8 |
|  |  |  |  |  |
| Self-reported sleep quality |  |  |  |  |
| Excellent/very good | 179 |  | 0.8 | 1.5 |
| Good | 431 |  | 0.9 | 1.8 |
| Less good/bad | 319 |  | 0.9 | 2.2 |
|  |  |  |  |  |
| Average hours of sleep last 4 weeks |  |  |  |  |
| <6 hours | 409 |  | 0.9 | 2.2 |
| 7 hours | 378 |  | 0.8 | 1.5 |
| >8 hours | 142 |  | 1.0 | 1.9 |
|  |  |  |  |  |
| MEQ-score |  |  |  |  |
| Definitely/moderately evening type (score 16-41) | 66 |  | 1.0 | 2.7 |
| Neither (score 42-58) | 621 |  | 0.9 | 1.8 |
| Definitely/moderately morning type (score 59-86) | 240 |  | 0.9 | 1.8 |
|  |  |  |  |  |
| Chronic diseases |  |  |  |  |
| None | 613 |  | 0.9 | 1.7 |
| >1 | 312 |  | 0.9 | 2.2 |

| Supplementary Table A2: Significant night work exposure-variables from main analyses with cardiometabolic risk factors included one at a time. Analyses are adjusted for age and education; analyses of night shift characteristics are restricted to current night workers. Estimates (Est.) are presented with their lower (L) and upper (U) 95% confidence intervals (CI). | | | | | | | | | | | | | | | | | | |
| --- | --- | --- | --- | --- | --- | --- | --- | --- | --- | --- | --- | --- | --- | --- | --- | --- | --- | --- |
|  | Adjustment for smoking | | | Adjustment for alcohol | | | Adjustment for physical activity | | | Adjustment for BMI | | | Adjustment for diastolic blood pressure | | | Adjustment for systolic blood pressure | | |
|  |  | | |  | | |  | | |  | | |  | | |  | | |
|  | Est. | L95%CI | U95%CI | Est. | L95%CI | U95%CI | Est. | L95%CI | U95%CI | Est. | L95%CI | U95%CI | Est. | L95%CI | U95%CI | Est. | L95%CI | U95%CI |
| Current work schedule | | | | | | | | | | | | | | | | | | |
| Permanent day | 1.00 |  |  | 1.00 |  |  | 1.00 |  |  | 1.00 |  |  | 1.00 |  |  | 1.00 |  |  |
| Shift work w/o night | 1.05 | 0.78 | 1.41 | 0.99 | 0.74 | 1.34 | 1.07 | 0.80 | 1.43 | 1.02 | 0.78 | 1.32 | 1.06 | 0.79 | 1.42 | 1.06 | 0.79 | 1.42 |
| Permanent night | **1.51** | **1.08** | **2.12** | 1.39 | 0.99 | **1.95** | **1.51** | **1.08** | **2.10** | 1.05 | 0.78 | 1.42 | 1.39 | 0.99 | 1.95 | **1.46** | **1.04** | **2.05** |
| Shift work w night | 1.15 | 0.94 | 1.39 | 1.13 | 0.93 | 1.38 | 1.16 | 0.95 | 1.40 | 1.06 | 0.89 | 1.26 | 1.15 | 0.95 | 1.40 | 1.15 | 0.95 | 1.40 |
|  | | | | | | | | | | | | | | | | | | |
| Number of night shifts per week | | | | | | | | | | | | | | | | | | |
| 1 | 1.00 |  |  | 1.00 |  |  | 1.00 |  |  | 1.00 |  |  | 1.00 |  |  | 1.00 |  |  |
| 2 | 1.20 | 0.98 | 1.47 | 1.18 | 0.96 | 1.45 | 1.17 | 0.96 | 1.43 | 1.00 | 0.83 | 1.20 | 1.16 | 0.95 | 1.42 | 1.17 | 0.95 | 1.44 |
| 3 | **1.37** | **1.01** | **1.85** | 1.31 | 0.97 | 1.78 | 1.31 | 0.97 | 1.76 | 1.02 | 0.77 | 1.33 | 1.34 | 0.99 | 1.80 | 1.35 | 0.99 | 1.82 |
| 4 or more | **1.51** | **1.10** | **2.07** | **1.40** | **1.01** | **1.93** | **1.48** | **1.08** | **2.02** | 1.13 | 0.85 | 1.50 | **1.38** | **1.01** | **1.89** | **1.46** | **1.06** | **2.01** |
|  | | | | | | | | | | | | | | | | | | |
| Number of consecutive night shifts | | | | | | | | | | | | | | | | | | |
| 1 | 1.00 |  |  | 1.00 |  |  | 1.00 |  |  | 1.00 |  |  | 1.00 |  |  | 1.00 |  |  |
| 2 | 1.21 | 0.90 | 1.62 | 1.16 | 0.86 | 1.55 | 1.18 | 0.88 | 1.58 | 1.05 | 0.81 | 1.36 | 1.15 | 0.86 | 1.53 | 1.18 | 0.88 | 1.59 |
| 3 | **1.53** | **1.16** | **2.04** | **1.47** | **1.11** | **1.95** | **1.55** | **1.17** | **2.05** | 1.23 | 0.95 | 1.58 | **1.47** | **1.11** | **1.94** | **1.49** | **1.12** | **1.98** |
| 4 or more | **1.47** | **1.07** | **2.02** | 1.37 | 0.99 | 1.89 | **1.46** | **1.07** | **1.99** | 1.08 | 0.81 | 1.43 | **1.40** | **1.02** | **1.91** | **1.42** | **1.04** | **1.95** |

| Supplementary Table A3: Mediation analyses of the association between work schedule characteristics and hsCRP, showing percent change estimates for the indirect effect via BMI (ACME), direct effect (ADE), total effect, and the proportion mediated. Estimates are presented as percent change in hsCRP (derived from log-transformed models) with 95% confidence intervals. Analyses are adjusted for age and education; analyses of night shift characteristics are restricted to current night workers. Estimates (Est.) are presented with their lower (L) and upper (U) 95% confidence intervals (CI). | | | | | | | | | | | | | | | | | |
| --- | --- | --- | --- | --- | --- | --- | --- | --- | --- | --- | --- | --- | --- | --- | --- | --- | --- |
|  |  | | | |  | | | |  | | |  | | |  | | |
|  |  | | | | Indirect effect (ACME)  Percentage change | | | | Direct effect (ADE)  Percentage change | | | Total effect  Percentage change | | | Mediated proportion | | |
|  |  | Total n | Ref.  n | Exposed n | | Est. | L95%  CI | U95% CI | Est. | L95% CI | U95% CI | Est. | L95% CI | U95% CI | Est. | L95% CI | U95% CI |
| Current work schedule | | | | | | | | | | | | | | | | | |
| Permanent day |  |  |  |  | |  |  |  |  |  |  |  |  |  |  |  |  |
| Shift work w/o night | 2vs.1 | 262 | 175 | 87 | | 2.0 | -12.3 | 18.9 | -0.2 | -22.3 | 27.5 | 1.8 | -23.6 | 36.6 | 28.3 | -542.9 | 594.3 |
| Permanent night | 3vs.1 | 235 | 175 | 60 | | **42.8** | **18.4** | **74.4** | -1.7 | -27.1 | 32.5 | 40.3 | -0.5 | 97.9 | 102.3 | -54.7 | 514.5 |
| Shift work w night | 4vs.1 | 782 | 175 | 607 | | 8.3 | -1.1 | 18.5 | 5.8 | -11.6 | 26.1 | 14.6 | -6.7 | 39.2 | 50.8 | -273.5 | 418.5 |
|  | | | | | | | | | | | | | | | | | |
| Number of night shifts per week | | | | | | | | | | | | | | | | | |
| 1 (1) |  |  |  |  | |  |  |  |  |  |  |  |  |  |  |  |  |
| 2 (2) | 2vs.1 | 500 | 272 | 228 | | **17.8** | **8.1** | **29.1** | 0.8 | -16.3 | 22.1 | 18.8 | -3.4 | 45.4 | 89.6 | -321.9 | 690.3 |
| 3 (3) | 3vs.1 | 346 | 272 | 74 | | **30.4** | **14.9** | **51.0** | 1.9 | -23.5 | 35.8 | 32.9 | -1.9 | 81.1 | 88.7 | -267.2 | 536.9 |
| 4 or more (4) | 4vs.1 | 338 | 272 | 66 | | **31.3** | **14.3** | **53.6** | 15.1 | -14.6 | 52.9 | **51.0** | **10.1** | **106.4** | **65.6** | **30.0** | **213.6** |
|  | | | | | | | | | | | | | | | | | |
| Number of consecutive night shifts | | | | | | | | | | | | | | | | | |
| 1 (1) |  |  |  |  | |  |  |  |  |  |  |  |  |  |  |  |  |
| 2 (2) | 2vs.1 | 274 | 91 | 183 | | 11.8 | -0.9 | 28.3 | 5.4 | -18.9 | 36.7 | 17.8 | -11.7 | 56.7 | 52.5 | -447.5 | 514.2 |
| 3 (3) | 3vs.1 | 334 | 91 | 243 | | **25.3** | **10.3** | **45.9** | 21.8 | -5.8 | 56.4 | **52.6** | **14.1** | **101.7** | **52.2** | **24.6** | **135.1** |
| 4 or more (4) | 4vs.1 | 214 | 91 | 123 | | **35.6** | **16.1** | **62.8** | 11.3 | -16.8 | 50.5 | **51.0** | **9.8** | **109.1** | **74.0** | **34.7** | **236.6** |

| Supplementary Table A4: The association between the number of weekly and consecutive night shifts and hsCRP concentration in shift workers with night work (exclusion of permanent night workers). Adjustment for age and education. | | | |
| --- | --- | --- | --- |
|  | Estimate | L95% CI | U95% CI |
| Number of night shifts per week | | | |
| 1 | 1.00 |  |  |
| 2 | 1.18 | 0.96 | 1.44 |
| 3 | 1.28 | 0.92 | 1.78 |
| 4 or more | **1.69** | **1.03** | **2.76** |
|  |  |  |  |
| Number of consecutive night shifts | | | |
| 1 | 1.00 |  |  |
| 2 | 1.17 | 0.87 | 1.56 |
| 3 | **1.48** | **1.11** | **1.96** |
| 4 or more | **1.41** | **1.001** | **1.99** |

| Supplementary Table A5: Mediation analyses of the association between work schedule characteristics and hsCRP, showing percent change estimates for the indirect effect via BMI (ACME), direct effect (ADE), total effect, and the proportion mediated. Estimates are presented as percent change in hsCRP (derived from log-transformed models) with 95% confidence intervals. Analyses are adjusted for age and education; analyses of night shift characteristics are restricted to current night workers with exclusion of permanent night workers. Estimates (Est.) are presented with their lower (L) and upper (U) 95% confidence intervals (CI). | | | | | | | | | | | | | | | | |
| --- | --- | --- | --- | --- | --- | --- | --- | --- | --- | --- | --- | --- | --- | --- | --- | --- |
|  |  | | | |  | | |  | | |  | | |  | | |
|  |  | | | | Indirect effect (ACME)  Percentage change | | | Direct effect (ADE)  Percentage change | | | Total effect  Percentage change | | | Mediated proportion | | |
|  |  | Total n | Ref.  n | Exposed n | Est. | L95%  CI | U95% CI | Est. | L95% CI | U95% CI | Est. | L95% CI | U95% CI | Est. | L95% CI | U95% CI |
| Number of night shifts per week | | | | | | | | | | | | | | | | |
| 1 (1) |  |  |  |  |  |  |  |  |  |  |  |  |  |  |  |  |
| 2 (2) | 2vs.1 | 498 | 272 | 226 | **17.6** | **7.5** | **29.3** | -0.2 | -17.0 | 20.2 | 17.4 | -4.2 | 43.2 | 92.9 | -469.7 | 712.8 |
| 3 (3) | 3vs.1 | 331 | 272 | 59 | **25.3** | **10.2** | **45.5** | -1.3 | -27.5 | 34.8 | 23.7 | -11.5 | 71.9 | 87.5 | -829.4 | 929.1 |
| 4 or more (4) | 4vs.1 | 295 | 272 | 23 | 21.0 | -0.1 | 49.3 | 43.1 | -9.3 | 123.7 | **73.2** | 7.4 to | 176.5 | 34.3 | -7.2 | 138.2 |
|  | | | | | | | | | | | | | | | | |
| Number of consecutive night shifts | | | | | | | | | | | | | | | | |
| 1 (1) |  |  |  |  |  |  |  |  |  |  |  |  |  |  |  |  |
| 2 (2) | 2vs.1 | 271 | 91 | 180 | 10.7 | -1.9 | 25.8 | 4.9 | -19.1 | 38.1 | 16.1 | -12.5 | 54.9 | 48.6 | -504. | 550.5 |
| 3 (3) | 3vs.1 | 316 | 91 | 225 | **22.9** | **7.9** | **41.6** | 21.6 | -6.4 | 58.3 | **49.4** | **12.4** | **99.1** | **51.4** | **21.7** | **144.7** |
| 4 or more (4) | 4vs.1 | 175 | 91 | 84 | **30.3** | **11.0** | **56.9** | 13.4 | -18.4 | 55.9 | **47.8** | **3.5** | **108.6** | **66.8** | **22.3** | **282.4** |
